# Activity of Laccase Immobilized on TiO_2_-Montmorillonite Complexes

**DOI:** 10.3390/ijms140612520

**Published:** 2013-02-11

**Authors:** Qingqing Wang, Lin Peng, Guohui Li, Ping Zhang, Dawei Li, Fenglin Huang, Qufu Wei

**Affiliations:** 1 Key Laboratory of Eco-Textiles, Ministry of Education, Jiangnan University, Wuxi 214122, China; E-Mails: wqq888217@126.com (Q.W.); ping1203.love@163.com (P.Z.); ldw19900323@163.com (D.L.); windhuang325@163.com (F.H.); 2 National Engineering Laboratory for Cereal Fermentation Technology & School of Biotechnology, Jiangnan University, Wuxi 214122, China; E-Mails: leogard2005@163.com (L.P.); leeanna101121@yeah.net (G.L.)

**Keywords:** montmorillonite, laccase, enzyme immobilization, TiO_2_

## Abstract

The TiO_2_-montmorillonite (TiO_2_-MMT) complex was prepared by blending TiO_2_ sol and MMT with certain ratio, and its properties as an enzyme immobilization support were investigated. The pristine MMT and TiO_2_-MMT calcined at 800 °C (TiO_2_-MMT800) were used for comparison to better understand the immobilization mechanism. The structures of the pristine MMT, TiO_2_-MMT, and TiO_2_-MMT800 were examined by HR-TEM, XRD and BET. SEM was employed to study different morphologies before and after laccase immobilization. Activity and kinetic parameters of the immobilized laccase were also determined. It was found that the TiO_2_ nanoparticles were successfully introduced into the MMT layer structure, and this intercalation enlarged the “d value” of two adjacent MMT layers and increased the surface area, while the calcination process led to a complete collapse of the MMT layers. SEM results showed that the clays were well coated with adsorbed enzymes. The study of laccase activity revealed that the optimum pH and temperature were pH = 3 and 60 °C, respectively. In addition, the storage stability for the immobilized laccase was satisfactory. The kinetic properties indicated that laccase immobilized on TiO_2_-MMT complexes had a good affinity to the substrate. It has been proved that TiO_2_-MMT complex is a good candidate for enzyme immobilization.

## 1. Introduction

Laccase (E.C.1.10.3.2) is a multicopper oxidase that catalyzes oxidation of various aromatic substrates with concomitant reduction of oxygen to water [1]. The low substrate specificity exhibited by laccase and its ability to oxidize many pollutants make it an ideal candidate for use in bioremediation [2], oxygen cathode in biofuel cells [3], biosensors [4], biobleaching, *etc.* However, some drawbacks have limited the use of free enzymes, such as instability, unrecoverable, and no direct electron transfer between free enzyme and bare electrodes. The immobilization of enzymes to some functional supports can not only increase the operational stability and durability, but also can endow it with some new properties, which may have synergy effects on pollutants treatment. Therefore, many efforts have been devoted to enzyme immobilization. A variety of supports with different functionality, morphology, and physical properties have been reported for the immobilization of enzymes such as polymer nanofibers [5,6], hydrogels [7], carbon nanotubes [8] and nanoparticles [9,10]. To evaluate the suitability of a material for enzyme immobilization the following factors are suggested to be important considerations: surface area to volume ratio, enzyme loading, flow rate, mass transfer, ease of separation, as well as reactor design [11]. In addition, selecting a reasonable support which may significantly improve the stability of immobilized enzyme should take the following factors into consideration: prevention of intermolecular interactions, enzyme structural rigidification, prevention of enzyme subunits dissociation, generation of artificial environments around the enzyme, enzyme reactivation, modulation of enzyme selectivity or specificity and reduction of inhibition problems [12].

Montmorillonite (MMT), consisting of a lamellar stack of crystalline, 1 nm thick aluminosilicate sheets, is one of the most commonly used enzyme supports. The properties of MMT can be tailored to meet different needs by simple methods such as acid activation, ion exchange, pillaring and intercalation with organic compounds [13]. Moreover, it has been realized that, by taking advantage of the high aspect ratio surface area of each layer within a multilayered structure, MMT may serve as a benign host to provide spacious sites for protein accommodation [14]. In recent years, MMT has been widely studied as a benign enzyme support. A. Naidja *et al.* [15] chose MMT coated with varying levels of hydroxyaluminum as the support, and investigated the effects of coating on adsorption, immobilization and activity of tyrosinase. Sanjay Gopinath *et al.* [13] studied the immobilization of three different kinds of enzymes including α-amylase, glucoamylase and invertase onto acid activated montmorillonite via two independent techniques—adsorption and grafting using glutaraldehyde as spacer, and proposed that the enzymes were situated at the periphery of the clay mineral particles whereas the side chains of different amino acid residues penetrated between the layers.

TiO_2_ has been widely used for the decomposition of a great variety of organic pollutants because of its particular advantages, such as high chemical stability, strong oxidizing power, low cost, nontoxicity, and so on. Especially in recent years, coupled photochemical and biochemical treatment has been highly focused on and proven to be more efficient for degradation of biorecalcitrant pollutants [16–18]. The objective of this study is to evaluate the immobilization ability of TiO_2_-MMT complexes by physical adsorption, which has been considered as a simple process since no toxic solvents are used [19]. To fully understand the effects of TiO_2_-MMT complexes on enzyme activities, we choose TiO_2_-MMT complexes calcined at 800 °C (identified as TiO_2_-MMT800) as well as pristine MMT for comparison. Due to the fact that the space between two adjacent layers will be broken by high temperature, the effect of interlayer could be ignored for TiO_2_-MMT800. The effects of pH, temperature and aging on the immobilized laccase were discussed. This kind of material could have great potential in pollution treatment.

## 2. Results and Discussion

### 2.1. The Structures of MMT and TiO_2_-MMT Complexes

**Figure 1:**
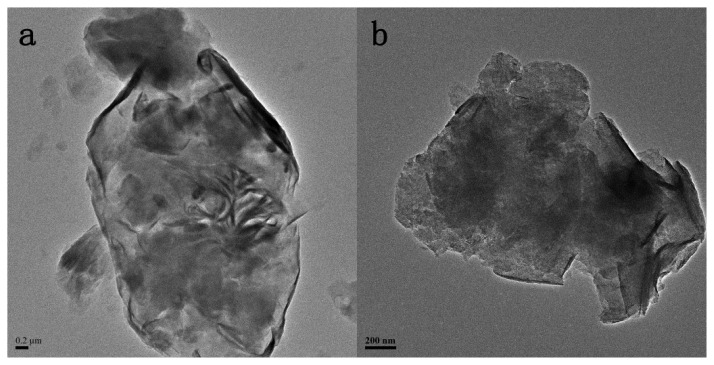

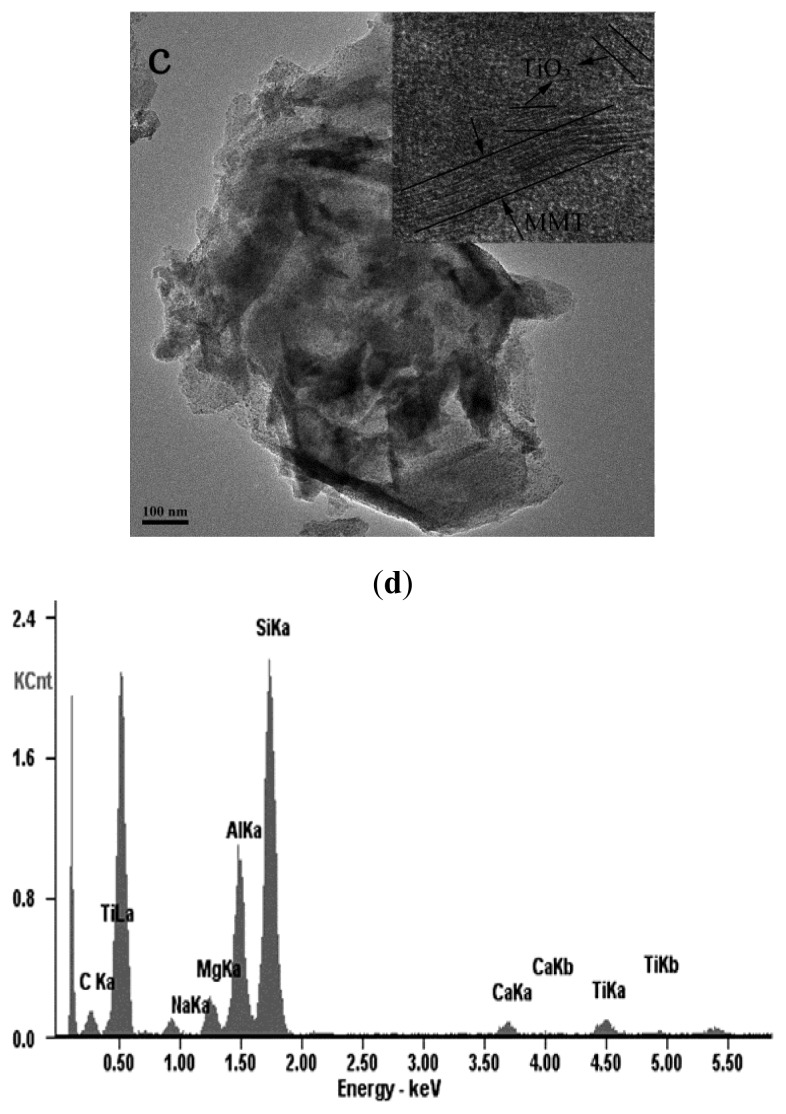
Transmission electron microscope (TEM) images of (**a**) pristine Montmorillonite (MMT); (**b**) TiO_2_-MMT; (**c**) TiO_2_-MMT800 and HR-TEM of TiO_2_-MMT800 (inset); (**d**) EDX of (c).

The TEM images reveal the internal structures of the pristine and the modified MMT nanohybrids as presented in Figure 1. The layered crystallites of MMT aggregate in large sized particles are shown in Figure 1a. As can be seen in Figs. 1b and 1c, there are numerous well-distributed small particles within the MMT layers, indicating well intercalation of TiO_2_ particles into the interlayers of MMT. It also needs to be noted that the small particles that existed in TiO_2_-MMT (Figure 1c) are not as clear as those in and the presence of a wide range of hydrolyzed species of Ti, such as monomeric TiO^2+^/Ti(OH)_2_^2+^ and polymeric species, in the TiO_2_ sol solution [20]. Figure 1d shows the EDX spectra of TiO_2_-MMT, indicating that the complex mainly consisted of Si, Al, Ti, Mg, Ca, Na, and further confirmed the presence of TiO_2_. Moreover, the inset image in Figure 1c clearly shows the lattice fringe of the TiO_2_ nanoparticles as well as the ordered MMT layers. In addition, the size of the TiO_2_ particles is less than 5 nm, because the dispersed layer silicates of MMT in this system behave as barriers to prevent TiO_2_ from agglomeration [20].

Introducing of TiO_2_ nanoparticles not only has effects on the surface morphology of MMT, but also changes its internal structure. As presented in Table 1, the thickness of adjacent MMT layer sheet is increased after interaction with TiO_2_ sol, and greatly decreased after the calcination process. Moreover, the BET data reveals the similar results as the d value derived from XRD, more specifically, the surface area are increased after exchange of CTAB with TiO_2_ sol, and decreased considerably after calcination. As is known, the thickness of two adjacent MMT layers without gallery heights is about 0.96 nm [21], indicating that the layer structure of TiO_2_-MMT800 has totally collapsed and no gallery heights existed between the layers.

### 2.2. Surface Morphology of the Nanohybrids before and after Enzyme Immobilization

**Figure 2:**
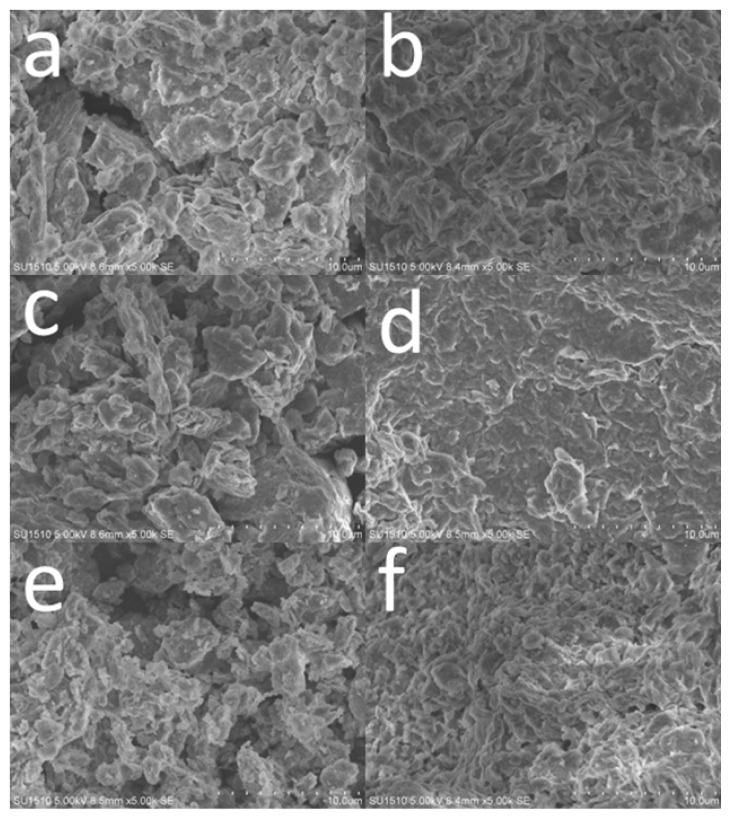
Scanning electron microscope (SEM) images of (**a**,**b**) pristine MMT, (**c**,**d**) TiO_2_-MMT, (**e**,**f**) TiO_2_-MMT800 before and after enzyme immobilization.

The morphology of the clays before and after enzyme immobilization was investigated by SEM, as shown in Figure 2. The pristine MMT (Figure 2a) clusters together with irregular shape, and the modified MMT before (Figure 2c) and after (Figure 2e) calcinations exhibits similar morphologies except that the diameters of the particles are decreased, which can be attributed to the dispersion effect during the preparation process of TiO_2_-MMT complexes. In general, the morphology of modified MMT is little affected by hydrolysis or intercalating process.

However, after enzyme immobilization, the surface morphologies show a significant change. The clays coated with adsorbed enzymes adhere to each other, as illustrated in Figure 2b,d,f. For pristine MMT (Figure 2b), the original structure can still be identified, while the other two samples (Figure 2d,e) show a compact and continuous structure after the enzyme immobilization, especially for TiO_2_-MMT complexes (Figure 2d).

So, it can be inferred that the existence of TiO_2_ enhanced the enzyme immobilization. In addition, the immobilized enzymes are mainly adsorbed on the surface of clays rather than intercalated in-between the clays, because TiO_2_-MMT800 also shows good immobilization capability though its layered structure is completely broken during the process of calcination under high temperature.

### 2.3. Kinetic Properties of Immobilized Enzyme

As for the enzyme immobilization capability of the three supports, the TiO_2_-MMT adsorbs more enzymes than that of the other two and the enzyme activity retention is also very good, as indicated in Table 1, which could be ascribed to the biofriendly microenvironment for the immobilized laccase created by the TiO_2_ sol on the upper surface or the interlayer in-between the clays.

The kinetic parameters of *K*_m_ and *V*_max_ are also presented in Table 1. *K*_m_ value is most useful in probing the ability of an enzyme to bind its substrate. An increase in *K*_m_ value for immobilized laccase is observed with ABTS as substrate. The result is in agreement with other investigators [22], indicating a lower affinity for the substrate caused by diffusional limitations and decreased protein flexibility after immobilization [23]. The *K*_m_ value for the immobilized enzymes on TiO_2_-MMT800 is the lowest among the three different supports, which signifies that the enzymes immobilized on TiO_2_-MMT800 is less distorted than the other two due to a lower interaction with the support and also a lower diffusion limitation. It is proposed by other reporters [13] that the enzymes are situated at the periphery of the clay mineral particles whereas the side chains of different amino acid residues penetrate between the layers. “*d* value” calculated from XRD results represents the interlayer distance of MMT. It can be inferred that for TiO_2_-MMT800, the space between two adjacent layers is completely destroyed by high temperature during calcination, as proved by the “d value” presented in Table 1. So, the enzymes immobilized on TiO_2_-MMT800 are mainly situated at the fringe of the MMT surface and no penetrating of side chains in-between the layers, which is considered to be the main reason of the lowest *K*_m_ value and the highest *V*_max_ value among the three different kinds of supports.

Therefore, TiO_2_-MMT complexes can be considered as a relatively better support for laccase immobilization.

### 2.4. The Distribution of Immobilized Laccase on MMT and TiO_2_-MMT Complexes

**Figure 3:**
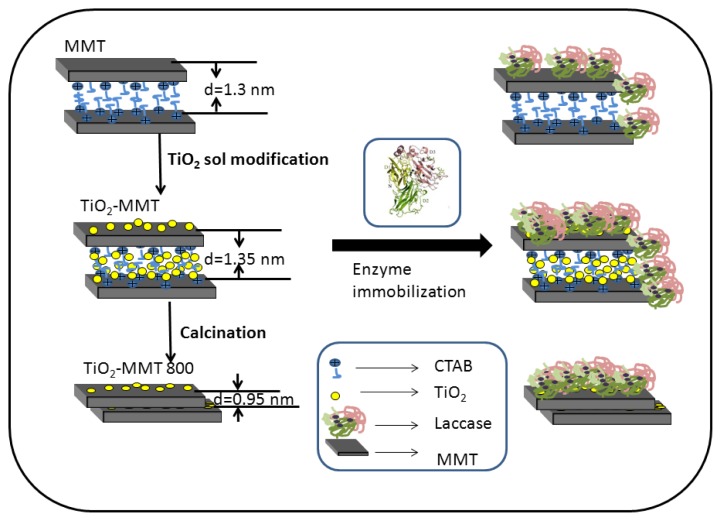
Schematic diagram of the enzyme immobilization mechanism.

A schematic diagram is proposed, as presented in Figure 3, showing the immobilization of laccase on the three different kinds of supports. For pristine MMT, the existence of CTAB enlarges the d-spacing, so the side chains of laccase will penetrate in-between the adjacent layer, leading to increased diffusion limitation during catalytic process. The intercalation of TiO_2_ sol further increases the d-spacing of MMT, and so the amount of enzymes with side chains penetrated into the MMT interlayer is increased. Though enzymes immobilized in this way will lose activity to some extent, the support can bind more enzymes in a larger quantity. In addition, the TiO_2_ nanoparticles situated on the upper layer of MMT can be ideal sites for the enzyme to locate; the same conclusion has been drawn in the literature [15]. So, TiO_2_-MMT adsorbs more enzymes than that of the other two supports, though the specific activity is not the highest. As for TiO_2_-MMT800, the MMT layered structure collapses during the calcination process, so an enzyme immobilized on TiO_2_-MMT800 is mainly due to the effect of surface entrapment. An enzyme immobilized in this way will maintain its initial activity to a great extent, while the drawback is that this kind of combination between enzyme and support is not very strong.

### 2.5. Effect of pH on Free and Immobilized Laccase

Changes in pH value have effects on the enzyme conformation and the dissociation degree of substrate and coenzyme, and thus affect the binding and catalysis between the enzyme molecules and substrate. Only at the specific pH value could the enzyme and substrate be most appropriately combined, and the optimum catalysis reaction occur.

**Figure 4:**
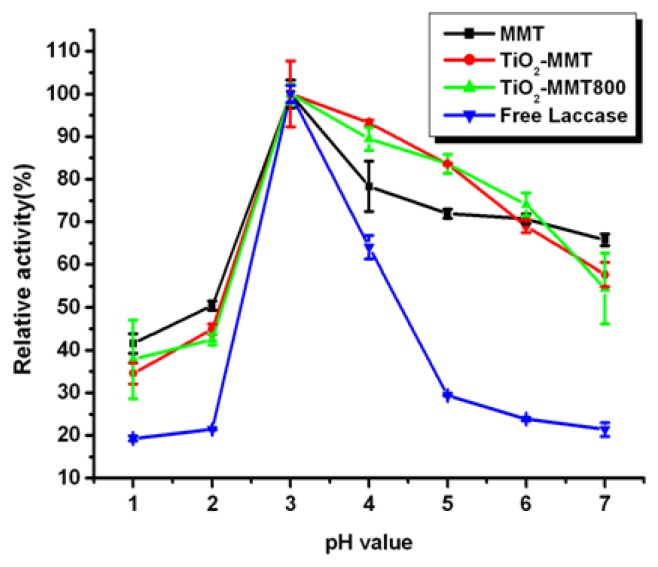
Effect of pH on the activity of free and immobilized laccase.

The free and immobilized laccases were incubated in buffers at 4 °C for 12 h with pH values ranging from 1 to 7. The immobilized laccase demonstrates much higher pH stability than free enzymes, especially in the pH range of 3–7, as indicated in Figure 4. This phenomenon can partially be attributed to the acid centers in MMT clay, that is, Lewis and Bronsted acid sites. Lewis acid is originated from the pillars structure of the pillared MMT, while Bronsted acid comes from the layered structure of pillared clay and the H^+^ released during the process of calcination. So, MMT can provide laccase with a relatively stable acid environment and improve its pH stability. In the range of pH 1–5, the pillared MMT shows better stability in the relatively alkali buffer (compared with optimum pH) and comparatively lower stability in the relatively acid buffer than the pristine MMT, which phenomenon can further confirm the effect of the Lewis and Bronsted acid sites in MMT clay.

However, in the pH range from 6 to 7, the results do not coincide with our previously suggested inference. So, there might be other factors which contribute to the pH stability as well. The enzyme immobilized by clay can be divided into two types, one is to be adsorbed on the surface of clay and the other is intercalated in-between its interlayers. According to the data presented in Table 1, pillared MMT shows better immobilization ability, from which it can be inferred that immobilized laccase mainly exist in the form of surface adsorption rather than intercalation. In addition, TiO_2_ has a good affinity for enzymes [24]. This means that the pH stability is also influenced by the existence state of TiO_2_, the structure of MMT as well as the way laccase bonded with the clay. The experimental results reveal that the immobilized laccase has higher activity stability across a broader pH range than the free laccase.

### 2.6. Effect of Temperature on the Activity of Free and Immobilized Laccase

**Figure 5:**
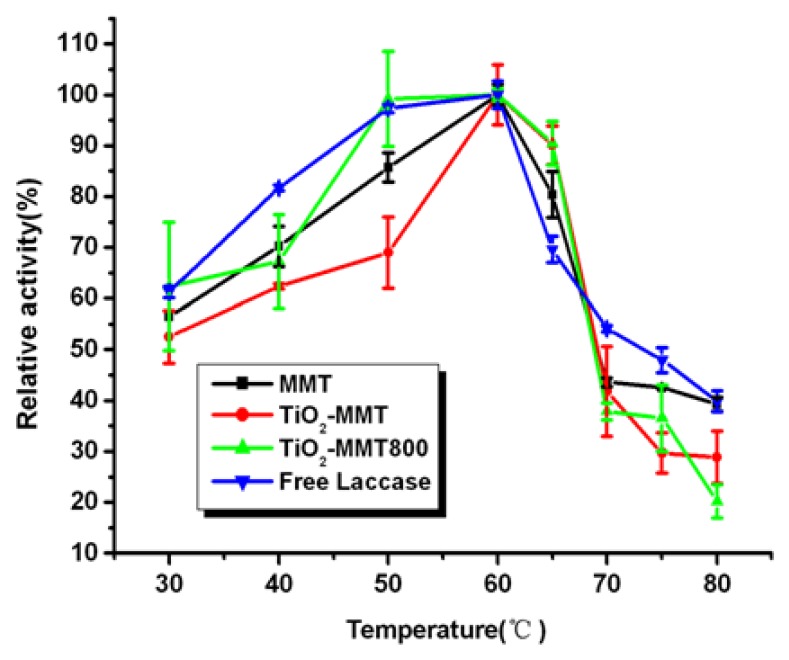
Effect of temperature on the activity of free and immobilized laccase.

The effect of temperature on relative activity of free and immobilized laccase is shown in Figure 5. As is known, MMT has been widely used as flame retardant material for its insulation property and favorable heat resistance property, which might lead to the displacement of the temperature-activity profile. The immobilized enzymes show a similar trend of temperature stability in the temperature range studied, and they keep high activity in the temperature range of 50 to 65 °C. However, at the lower temperature range (30–50 °C) and the slightly higher temperatures (65–80 °C), the relative activity loss of immobilized laccase is higher than the free one.

### 2.7. Storage Stability

**Figure 6:**
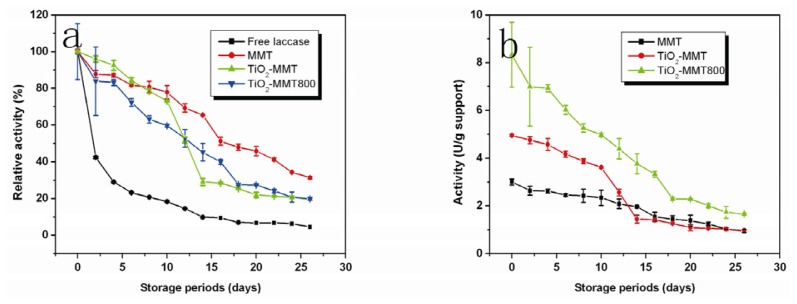
Effect of aging on (**a**) relative activity and (**b**) specific activity.

For enzyme immobilization, storage stability is one of the most important parameters to be considered, because it can greatly affect the activity retention during the storage. The stabilities of the immobilized laccase were compared under the predetermined conditions in sodium acetate buffer (100 mM, pH 4.5) at room temperature. As is shown by Figure 6a, the activity retention of the enzyme immobilized on the pristine MMT, TiO_2_-MMT and TiO_2_-MMT800 after 20 days is 45%, 21% and 27%, respectively, indicating that the enzyme immobilized on pristine MMT shows the best storage stability under the same storage conditions. It can also be observed that during the first 10 days, the activity loss of enzyme immobilized on TiO_2_-MMT800 is more than those of the other two, because the enzyme is just adsorbed on the surface of the TiO_2_-MMT800 and not as firmly bonded as those immobilized on MMT and TiO_2_-MMT supports, which have certain interlayer space for side chains of enzymes to penetrate.

As seen in Figure 6b, the initial activities of the laccase on pristine MMT, TiO_2_-MMT and TiO_2_-MMT800 are 3.004, 4.947 and 8.329 U, respectively. The activity of the immobilized laccase on TiO_2_-MMT800 is about 2–3 times higher than those of immobilized laccase on MMT and TiO_2_-MMT, which is also in agreement with the results shown in Table 1. The high initial activity of laccase immobilized on TiO_2_-MMT800 can be well explained by the supports’ high enzyme immobilization ability and enzyme activity retention, as indicated in Table 1. Therefore, though the immobilized laccase on the pristine MMT shows better storage stability, the enzyme activity of immobilized laccase on TiO_2_-MMT complexes is higher.

## 3. Experimental Section

### 3.1. Materials

Laccase (EC 1.10.3.2) was provided by Novozymes (Guangzhou, China). 2,2′-Azino-bis-(3-ethylbenzothiazoline-6-sulfonic acid) (ABTS) was obtained from Shanghai Richu Biosciences Co., Ltd. (Shanghai, China). The organically modified montmorillonite (O-MMT) by hexadecyl trimethyl ammonium bromide (cation exchange capacity, CEC, 97 mequiv./100 g of clay) was purchased from Zhejiang Fenghong Clay Chemicals Co., Ltd. (Huzhou, China). The polyacrylonitrile (PAN, M_w_ = 79,100) powder was obtained from Aldrich (Shanghai, China). The 99.5% *N*,*N*-dimethyl formamide (DMF), tetrabutyl titanate [Ti(OC_4_H_9_)_4_, CP], ethanol (EtOH, AR) and hydrochloric acid (HCl, AR) were all used as received.

### 3.2. Preparation of TiO_2_–MMT Complexes

The TiO_2_ sol was prepared by the hydrolysis of Ti(OC_4_H_9_)_4_ in the blended solution of deionized water and HCl. The detailed process was described in our previous research [25].

One gram MMT powders were added to the TiO_2_ sol with the volume of 50 mL and vigorously stirred for 3 h at room temperature and aged for 24 h. Subsequently, the MMT suspensions were centrifuged and filtered. The obtained wet cakes were dried under vacuum at 80 °C and then calcined at 800 °C in air for 2 h, identified as TiO_2_-MMT800, while the uncalcined as TiO_2_-MMT.

### 3.3. Laccase Immobilization by Physical Adsorption

Pristine MMT and TiO_2_-MMT complexes were used as the supports. For a typical immobilization process, 100 mg supports were mixed with equal volumes (80 mL) of enzyme solution (3 g/L) dissolved in 100 mM sodium acetate (pH = 4.5) buffer and magnetically stirred for 5 h in an ice bath. Then the supports were filtered and washed several times with buffer until no laccase activity was detected in the washing buffer.

### 3.4. Structural Characterization

The Brunauer-Emmett-Teller (BET) specific surface areas of the pristine MMT and modified MMT were obtained using a ASAP 2020 instrument (Micromeritics, Norcross, GA, USA). The samples were degassed at 115 °C for 8 h before the measurement.

The X-ray diffraction (XRD) experiments were carried out in a Philips MPD-18801 diffractometer (BRUKER AXS GMBH, Beijing, China) using Cu Kα (λ = 0.15406 nm) radiation and a fixed power source (40 kV, 40 mA).

The structures of the composite nanoparticles was evaluated from TEM micrographs obtained by a transmission electron microscope (TEM) (JEOL2100, Tokyo, Japan) operating at 80 kV and characterized by a point-to-point resolution.

Scanning electron microscope (SEM, Quanta 200, Holland FEI Company, Beijing, China) was used to investigate the surface morphology of composite nanoparticles before and after enzyme immobilization.

### 3.5. Protein Concentration and Laccase Activity

#### 3.5.1. Protein Concentration

The protein content in the immobilized supports was determined by subtracting the protein of the supernatant after immobilization from the initial enzyme solution, using Bradford’s method. To be more specific, the protein concentration was measured by the absorbance of the Coomassie brilliant blue (CBB) at 595 nm, with bovine serum albumin as the protein standard.

#### 3.5.2. Activity Assay of Free and Immobilized Laccase

The activity of free and immobilized laccase was determined according to the method reported by Lu *et al.* [23] with some modifications. The reaction mixture was composed of 0.5 mM ABTS, 100 mM sodium acetate buffer (pH = 4.5) and a suitable amount of free and immobilized laccase. The reaction was started by adding 0.1 mL 15 mM ABTS into 2.9 mL sodium acetate buffer containing certain amount of free or immobilized laccase. For immobilized laccase, the supernatant used for activity assay was obtained by centrifugation at 12,000 rpm for about 40 s, and the incubation time and the centrifugation time were controlled to be altogether 3 min.

### 3.6. Kinetic Parameters

Kinetic tests were carried out at 25 °C in 100 mM sodium acetate (pH = 3.0) buffer using ABTS as the substrate, with the substrate concentration varied from 0.1 to 1 mM.

The kinetic parameters of *K*_m_ and *V*_max_ were calculated according to the Lineweaver-Burk double reciprocal models as follows:

$$1/v=(K_{m}/V_{\text{max}})(1/S)+1/V_{\text{max}}$$

where *v* is the initial catalytic rate and *S* is the substrate concentration.

### 3.7. Effect of Temperature and pH on the Activity of Immobilized and Free Enzyme

To determine the resistance to pH changes, activities of the free and immobilized enzymes were determined by measuring the activities after they were put in different buffer solutions (pH 1–7) for 12 h, while the effect of temperature on the activity of free and immobilized laccase was determined by measuring the enzyme activity over the temperature range of 30–80 °C at pH = 4.5.

### 3.8. Storage Stability

The stability of immobilized enzyme was determined by calculating their activity retention ratio during storage at 4 °C in 100 mM sodium acetate buffer solution (pH 4.5), at regular intervals up to 26 days.

## 4. Conclusions

TiO_2_-MMT complexes were successfully fabricated and proved to be better supports than the pristine MMT for laccase immoblization. The amount of enzyme adsorbed by TiO_2_-MMT was higher than those of the other two supports, while the enzyme immobilized on TiO_2_-MMT800 showed better kinetic properties and the activity retention was as high as 88%. After immobilization, the pH stability was greatly improved, while no significant improvement could be observed for temperature stability. Although the storage stability of laccase immobilized on TiO_2_-MMT complexes was not as good as the pristine MMT, the activity displayed by TiO_2_-MMT complexes was higher than that of pristine MMT. Laccase immobilized on TiO_2_-MMT support combined the advantages of biocatalytic and photocatalytic properties as well as high adsorbability, so this kind of material is believed to be ideal candidate for waste treatment.

## Figures and Tables

**Table 1:** Typical characteristics of three different supports, and adsorption capacity, activity as well as kinetic parameters for free and immobilized laccase.

Sample	*d* (nm)	Langmuir surface area (m^2^/g)	Adsorbed protein (mg.g^−1^)	Specific activity (U.mg^−1^)	Activity retention (%)	*K*_m_ (mM)	*V*_max_ (μmol/mg protein min)
Free laccase				5.657	100	0.257	62.112
Pristine MMT	1.299	77.049	27.178	2.951	52.16	5.706	4.931
TiO_2_-MMT	1.355	99.003	35.896	4.227	74.72	3.493	5.548
TiO_2_-MMT800	0.952	32.128	33.332	5.004	88.46	1.303	7.010
